# Use of Electric Discharge for Simultaneous Control of Weeds and Houseflies Emerging from Soil

**DOI:** 10.3390/insects11120861

**Published:** 2020-12-03

**Authors:** Yoshinori Matsuda, Kunihiko Shimizu, Takahiro Sonoda, Yoshihiro Takikawa

**Affiliations:** 1 Laboratory of Phytoprotection Science and Technology, Faculty of Agriculture, Kindai University, Nara 631-8505, Japan; 2 Mikado Kyowa Seed, Co. Ltd., Chiba 267-0056, Japan; kunihikoshimizunozomi@yahoo.co.jp; 3 Sonoda Seisakusho Co. Ltd., Osaka 547-0006, Japan; sonoda@ace.ocn.ne.jp; 4 Plant Center, Institute of Advanced Technology, Kindai University, Wakayama 642-0017, Japan; takikawa@waka.kindai.ac.jp

**Keywords:** housefly, arc-discharge exposure, weed control, insect pest control, dynamic electric field, greenhouse organic farming

## Abstract

**Simple Summary:**

In greenhouse organic farming, soil fertilized with cattle manure frequently harbors weeds and houseflies that result in difficulties for pesticide-free cultivation. The purpose of this study was to devise an electrostatic apparatus to control weeds and houseflies emerging from ground soil simultaneously using electric-field-based techniques. For weed eradication, several identical iron plates were placed in parallel at a predefined interval and negatively charged. Due to their conductive nature, plant shoots emerging from the soil between the plates in the apparatus were subjected to an arc discharge from the charged plate when the rhizosphere soil was electrified. Houseflies emerging from underground pupae were controlled using a similar electrostatic principle; flies that entered the space between the negatively charged and grounded iron plates of the second apparatus were exposed to arc discharge. A practical apparatus was constructed by combining two apparatuses devised to control weeds and houseflies, then surveyed for its functionality in a greenhouse environment. The apparatus remained functional during continuous operation in a greenhouse environment, indicating that the method is a promising tool as a pesticide-alternative approach in organic farming.

**Abstract:**

An electrostatic apparatus was developed to control weeds and houseflies emerging from ground soil in a greenhouse simultaneously. Identical iron plates were placed in parallel at a defined interval and fixed in an iron frame. Two sets of fixed iron plates were used, one for weed control and one for fly control. For weed control, all of the iron plates were negatively charged, and negative charges accumulated on the plates were released to weed shoots through arc discharge. Houseflies were introduced into the space between the negatively charged and grounded plates, then subjected to arc discharge from the charged plates. Both plant shoots and adult houseflies are electrically conductive; thus, they were killed by discharge-exposure in the electric field between the charged iron plate and the ground soil, and between the charged and grounded plates, respectively. In practical use, these two devices were assembled as a two-level apparatus for simultaneous control of both targets. Several apparatuses were linked together, which increased the total electricity charge on the plates and produced a stronger discharge force sufficient to kill all targets. Thus, this study provides an electrostatics-based pest-control method for pesticide-independent greenhouse farming.

## 1. Introduction

The introduction of animal and green manure or food waste compost into soil beds in a greenhouse is a routine approach to soil fertilization in organic farming. The major organic fertilizer in greenhouse cultivation is cattle manure, which is typically introduced into soil beds once or twice each year. Nevertheless, the use of manure-fertilized soil results in serious challenges; cattle manure frequently contains weed seeds and/or housefly (*Musca domestica*) larvae and pupae, resulting in the appearance of weed shoots and adult houseflies during plant cultivation. In an organic cultivation system, the presence of weeds in soil beds requires manual weeding, because no herbicides are used. Additionally, the emergence of houseflies poses a potential risk to public health. Food poisoning caused by *Escherichia coli* O157 occurs frequently in people who have eaten fresh food materials contaminated by this pathogen. *E. coli* O157 originally lives in the intestines of cattle and sheep, in which it does not cause disease, and spreads to the human food chain through feces from these animals [1,2,3]. Importantly, this bacterial pathogen has been transferred from cattle manure used for soil fertilization [4]. Houseflies have been identified as the vector for pathogen dissemination [2,3]. To solve these weed and housefly problem, soil beds were covered with mulch. However, the application of mulch is unsuitable for plant cultivation, especially during the summer, because it considerably increases soil temperatures. Thus, the purpose of this study was to develop a physical method to resolve these weed and housefly problems.

In our efforts to promote organic farming of greenhouse tomatoes, we have developed various pesticide-free measures to control all classes of pests (pathogens, insects, and weeds). One approach to insect and pathogen control has been to use an electric field screen at the openings of the greenhouse to prevent entry of airborne spores of fungal pathogens and flying insects, which pass through conventional, supposedly insect-proof, nets [5,6,7,8]. The electric field screen is used to create an air-permeable electric field barrier and to capture spores and insects that enter the electric field [9]. A bamboo-blind-type electric field screen, a simple and inexpensive version of an electric field screen, was developed for a plastic hoop greenhouse on a small-scale farm [10]. Electrostatic insect sweepers [11] and electrostatic flying-insect catchers [12] are portable apparatuses that have been used to capture insects on plant leaves and over plants in a greenhouse, respectively. Biological measures have been a supplementary approach to insect control; a successful approach comprises the application of entomopathogenic phylloplane bacteria, which are epiphytic bacteria that stably colonize tomato leaves, for biocontrol of phytophagous ladybird beetles. Notably, the ladybird beetles that ingest bacteria-treated leaves are quickly killed due to robust bacterial multiplication inside the insect body [13,14,15]. Thus, using a systemic combination of physical and biological measures to control pests, it is possible to harvest fruits and vegetables grown without pesticides.

In the present study, we designed a novel electrostatic instrument to kill two types of targets (weed shoots and houseflies) emerging from the ground selectively, using electrostatic principles. The instrument consists of identical metal plates placed in parallel and provided with opposing charges to form a dynamic electric field between the plates. This electric field is the site of pest extermination; the target is subjected to a high-voltage electric current through discharge, because the conductivity of the target is higher than that of the air between the poles. As a result of the strong impact of the arc discharge, the growing point of a weed shoot is destroyed, while flies are killed instantaneously. In this study, we first developed an electrostatic instrument for weed eradication, then modified it for possible application to fly control. Based on the results, we discuss the feasibility of the present instrument for simultaneous control of weeds and insects.

## 2. Materials and Methods

### 2.1. Plant Species

Barley (*Hordeium vulgare* cv. Kobinkatagi), oat (*Avena sativa* cv. Negusaredaigi), soybean (*Glycine max* cv. Natsunokoe), tomato (*Solanum lycopersicum* cv. Momotaro fight), watermelon (*Citrullus lanatus* cv. Tahiti), and sunflower (*Helianthus annuus* cv. Konatsu) were used as model plants for graminaceous (both barley and oat), leguminous, solanaceous, cucurubitaceous, and asteraceous weeds, respectively. Germinated seeds of these plants were sown in soil pots or a seedbed in a greenhouse, and newly emerged shoots were used in the following experiments.

### 2.2. Test Insects

Adult houseflies (*Musca domestica* Linnaeus) were used in this study. Housefly pupae were purchased from Sumika Technoservice (Hyogo, Japan) and maintained in a growth chamber (25.0 ± 0.5 °C 12-h photoperiod at 4000 lux) until new adults emerged. Newly emerged adults were collected with an insect aspirator and used for experiments. The body sizes of the adult houseflies were measured using 30 adult test insects collected randomly, revealing the following characteristics: 8.3 ± 1.1 mm (from head to wing edge), 3.1 ± 0.7 mm (in thickness), and 3.9 ± 0.6 mm (in width).

### 2.3. Assay of Soil Electric Conductivity

In the first experiment, to examine the effect of water loss on changes in the electric conductivity of the soil, the soil water content was measured by the loss-on-drying method [16]. Soil from the greenhouse seedbed was placed in a container and weighed, then dehydrated in a convection oven at 30 °C. At predefined intervals, the soil was removed from the oven for re-weighing. This procedure was continued until the soil weight remained constant. Finally, the difference between the initial and final weights was calculated to determine the quantity of moisture (soil water) removed. Based on the weight-loss calibration curve, soil samples containing different proportions of water were used for experiments.

**Figure 1:**
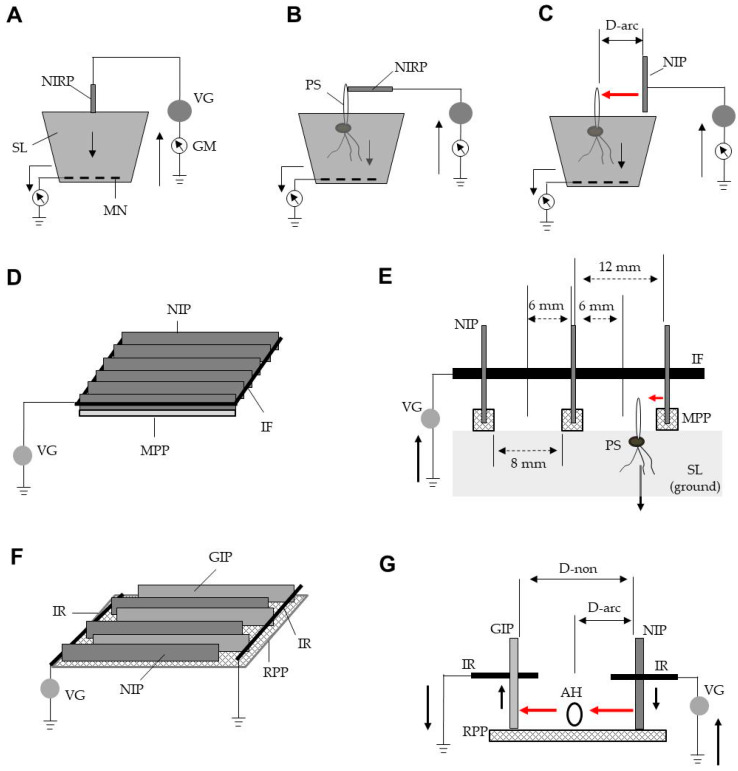
(**A**–**C**) Methods for examination of soil electric conductivity, examination of plant shoots grown in soil, and exposure of plant shoots to arc discharge, respectively. (**D**) Configuration of an electrostatic weed-eradicator (EWE). (**E**) Cross-sectional view of the EWE. (**F**) Configuration of an electrostatic insect-eradicator (EIE). (**G**) Cross-sectional view of the EIE. Solid arrows represent the movement of electricity, while red arrows show aerial movement of electricity through arc discharge. Abbreviations: NIRP, negatively charged iron rod probe; SL, soil; MN, metal net; VG, voltage generator; GM, galvanometer; PS, plant shoot; NIP, negatively charged iron plate (conductor); IF, iron frame; MPP, mortised polypropylene plate (insulator); GIP, grounded iron plate (conductor); IR, iron rod; RPP, reticulate polypropylene plate (insulator); AH, adult housefly; D-non, distance causing no arc discharge; D-arc, distance causing arc discharge.

Soil with various water contents was placed in pots, the bottoms of which were covered with a stainless-steel mesh linked to a grounded line. A probe consisting of an iron rod (length, 5 cm; diameter, 5 mm) that had been negatively charged by a direct-current voltage generator (maximum current, 10 mA; maximum voltage, −12 kV; Max Electronics, Tokyo, Japan) was placed in contact with the soil surface (Figure 1A). The magnitude and direction of electricity flow were recorded using a galvanometer (Sanwa Electric Instrument, Tokyo, Japan) that had been integrated into the grounded line. The conductivity of the test plant shoots was measured by touching them directly on the iron probe, while the electric current was confirmed by using the galvanometer (Figure 1B). In this experiment, soil with a water content of more than 80% was used to ensure sufficient conductance.

### 2.4. Exposure of Plant Shoots to Arc Discharge and Construction of an Electrostatic Weed Eradicator (EWE)

Figure 1C demonstrates the method used for aiming electricity accumulated on the charged plate (size, 1 × 30 cm^2^; thickness, 1 mm) at plant shoots via arc discharge. In this experiment, a pot containing soil with plant shoots was watered until the excess water leaked through the hole in the bottom of the pot; when the flow stopped, the soil and shoots were used for the experiment. The plate was brought close to the shoot in a stepwise manner, then charged with the highest voltage (−12 kV) of the voltage generator to investigate the occurrence of arc discharge between the plate and the shoot at each test interval. The sound produced by the arc discharge was measured in decibels using a sound-level meter (Sato Tech, Kanagawa, Japan). The sound profile was recorded with a spectrum analyser integrated into the sound-level meter. Simultaneously, the current profile was recorded using the current detector integrated into the voltage generator. Discharge-exposed shoots were photographed with a thermographic camera (Flir One; FLIR Systems, Wilsonville, OR, USA) to compare heat-zone images among discharge-exposed and non-exposed samples. The temperatures of shoot apical areas were determined with the multiple spot temperature meters for selectable onscreen temperature tracking regions in the camera. The subsequent growth of discharge-exposed shoots was recorded for 1 week to determine the degree of wounding of the discharge-exposed shoot apical regions. These shoots were classified into two types (NE- and RE-shoots): NE-shoots exhibited a lethal wound that resulted in no subsequent elongation, while RE-shoots resumed subsequent elongation (a non-lethal wound). Furthermore, RE-shoots were exposed to discharges under the same conditions continuously until they did not elongate. Plant shoot exposures to discharges were video-recorded.

Figure 1D,E shows the configuration of the EWE, which consisted of identical iron plates, a voltage generator, and mortised polypropylene square rods (width of one side, 5 mm; length, 30 cm). The bottom sides of the iron plates were mortised with a polypropylene rod to insulate them from the soil. The plates were arrayed in parallel at a predefined interval (12 mm) and welded to an iron frame. The iron frame was linked to a voltage generator.

### 2.5. Exposure of Flies to Arc Discharge and Construction of an Electrostatic Insect Eradicator (EIE)

In a preliminary experiment, the two iron plates described above were placed in parallel at an interval of 12 mm, then linked to a direct current voltage generator and a grounded line, respectively. The voltage was raised gradually to determine the range (−1 to −10 kV) of voltage that did not result in mechanical discharge (arc discharge between the plates) in the absence of houseflies.

In the first experiment, the plates were charged with different voltages that did not result in mechanical discharge. Adult houseflies were individually propelled into the space between the plates to examine the occurrence of arc discharge from the charged plates to the flies at a given voltage. The intensity of the arc-discharge sound was recorded by the method described above. The survival of discharge-exposed flies was assessed both immediately and at 6 h after discharge exposure to distinguish between flies that died instantaneously and those that died after a short delay. Flies walking or flying at the time of assessment were considered non-damaged flies. Exposure of adult houseflies to arc discharge was video-recorded.

In the second experiment, the EIE was constructed to expose flies to arc discharge upon entry to the electric field (Figure 1F,G). Several iron plates were arrayed in parallel at an interval of 24 mm and welded to an iron rod. Of the two identical sets of the welded plates, one was linked to a voltage generator, while the other was linked to a grounded line. The iron rods of the two sets were alternately arranged in parallel at an interval of 12 mm with a comb-shape polypropylene spacer, then placed vertically on a reticulate insulator plate (thickness, 1 mm; mesh size, 10 mm) and fixed with a polypropylene frame.

### 2.6. Combination of the EIE with the EWE to Control Weeds and Flies Simultaneously

**Figure 2:**
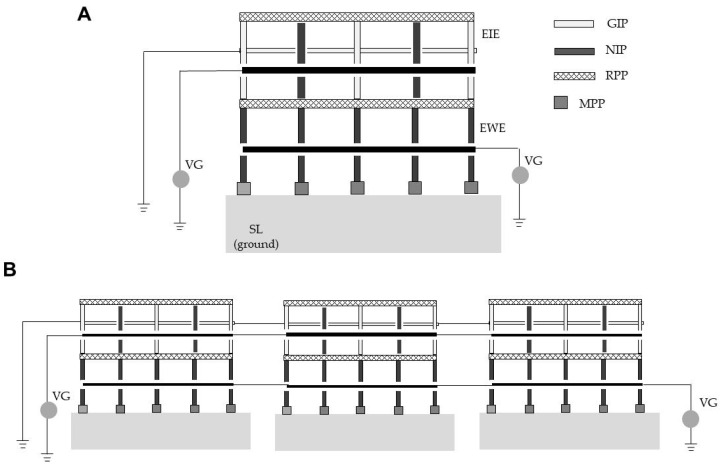
Diagrams of a combined apparatus (EIE/EWE) for simultaneous control of weeds and flies emerging from soil (**A**) and triplicate EIE/EWEs (**B**). Abbreviations are given in the legend of Figure 1.

To control weeds and flies emerging from soil simultaneously, we combined two apparatuses to construct a two-level apparatus (EIE/EWE) (size, 30 × 30 cm; 23 iron plates per apparatus) (Figure 2A). The EIE/EWE exposed weeds to arc discharge at the lower level and houseflies to arc discharge at the upper level.

In the first experiment, two to five EIE/EWEs were linked together with an electric wire as shown in Figure 2B, then charged with −10 kV. A single EIE/EWE apparatus was used in a similar manner. Adult houseflies were propelled into the space between the plates of one EIE/EWE selected at random. The arc-discharge sound was recorded by the method described above. The discharge-exposed flies were collected and examined for survival both immediately and at 6 h after discharge exposure. In subsequent experiments, triplicate EIE/EWEs were used (Figure 2B).

In the second experiment, triplicate EIE/EWEs were placed on three seedbeds (sites A–C) and linked together, as shown in Figure 2B. Housefly pupae were individually placed on insulator dishes (the white polystyrene cap of a small vial) to avoid the effects of electric conduction of the soil. Thirty caps with a pupa were placed at random on the soil between the plates of three EIE/EWEs (10 pupae per apparatus). Ninety seeds of the test plants described above (five seeds per species) were sown at different depths in the soil between the plates of the EIE/EWEs (30 shoots per apparatus). The EIE/EWEs were continuously charged with −12 kV (EWEs) and −10 kV (EIEs) for 1 month. The seedbeds used were typically watered twice per day. The soil inside the apparatus was carefully supplied with water drops from a pipette, three times per day, to avoid placement of water drops on the pupa dish. Shoot elongation and adult fly emergence from pupae were recorded daily during the experimental period. Dead flies were collected during each watering session. At the end of the experiment, the number of seeds that had developed shoots and the number of inactive pupae (from which adult flies had not emerged) were counted. In this experiment, the mean number of days required for shoots to enter the electric field and be killed was determined. The mean number of days for pupae to become adult flies and be killed during discharge exposure was also recorded.

### 2.7. Statistical Analysis

All experiments were repeated five times, and data are presented as the mean and standard deviation. Analyses were performed to identify significant differences among conditions, as well as correlations among factors, as shown in the figure and table legends.

## 3. Results and Discussion

Discharge is defined as electric current generation between opposite poles due to the dielectric breakdown of gases in the electric field according to the potential difference between the opposite poles [17]. If the grounded conductor is one of the poles (i.e., the recipient of electricity), the discharge occurs more easily, as this conductor receives electricity without any restriction (in this experiment, 10 mA of the maximum current of the voltage generator). In an electric field, a corona discharge first occurs and changes from a glow discharge (or surface discharge) to a brush-like discharge as the applied voltage increases and/or the distance between the poles decreases; the discharge breaks down with the occurrence of an arc discharge between the two poles [18].

Plants are conductive, such that on receiving electricity resulting from the discharge of a charged conductor the electric current flows through their bodies [19]. Arc discharge can produce a continuous electric current that causes damage to targets due to heating, based on the Joule effect [20]. In addition, arc discharge produces a strong force that can destroy small organisms through a high-voltage-mediated transient electric current flow [21,22].

In this study, the first target was weeds that emerged from soil. The apparatus designed in this study produces an electric circuit. Namely, the electricity is pumped upwards from a grounded conductor using the voltage produced by a voltage generator. It then accumulates on the surface of the iron plate connected to the voltage generator, and is sent back to the grounded conductor through the plant shoot growing in the soil. However, the release of electricity from the charged plate is impeded by the air between the plate and the plant. Therefore, a relatively high force (i.e., a high potential difference) to break down the resistance of the air (i.e., dielectric breakdown of gases) is essential for successful transfer of the electricity [17]. This can be achieved by an application of a higher voltage to the plate and/or a reduction of the distance between the charged plate and the plant. Soil conductivity is an additional impediment to current flow. Plants growing in soil are electrically grounded. However, if the soil becomes less conductive, the plants cannot receive discharge from charged conductors, even if other conditions are satisfied. In the preliminary experiment, we observed that soil conductivity was frequently reduced due to dryness. Subsequently, we focused on the conductivity of rhizosphere soils to ensure successful current flow in the plant body.

### 3.1. Soil Electric Conductivity Is a Basic Requirement for Electric Discharge Treatment of Plant Shoots

**Figure 3:**
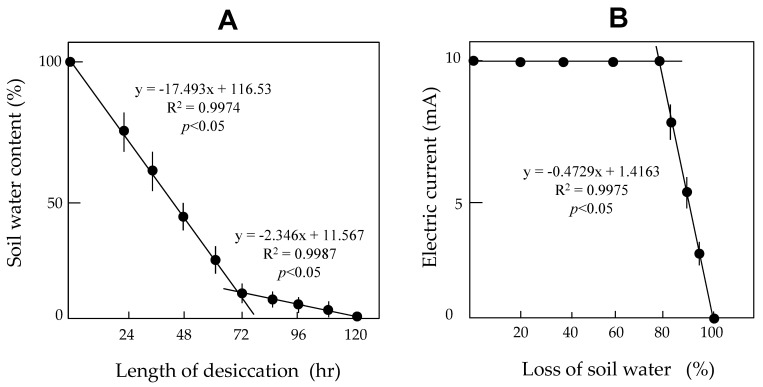
Relationships between duration of desiccation and loss of soil water by the loss-on-drying method (**A**) and changes in electric conductivity of soil samples with different degrees of water loss (**B**). Five soil samples were used for each duration of desiccation and each degree of water loss. Two regression lines are shown in each figure.

In the first experiment, we examined the relationship between changes in soil conductivity and loss of soil water content. The water content of soil was determined by thermogravimetric analysis (loss-on-drying method). This method is simple and the results are highly reproducible. Figure 3A shows the temporal change in test soil weights. The technique used in this study effectively dehydrated the soils to desired levels by changing the duration of desiccation. The data exhibited a high degree of reproducibility. There were two distinct phases in the loss of soil water: an initial rapid loss and a slower second phase. In both phases, a linear relationship was observed between the duration of soil desiccation and the extent of soil water loss. The total water content constituted 35–40% of the soil weight. Water may also become locked in molecular structures as bound moisture, such that greater amounts of heat energy are needed to release the tightly bound moisture [23]. Our loss-on-drying treatment likely promoted vaporization of free water in the soil.

Using this method, we collected soil samples with different degrees of water loss and examined their electric conductivities. We found that the conductivity did not change until 80% of the water content had been vaporized, and then drastically decreased at greater degrees of water loss (Figure 3B). These results indicate that for our technique, water loss from the soil should be maintained at less than 80% of total water, to ensure sufficient electric conductivity of the soil. Notably, the current-detection assay using the direct-touch method for plant shoots (Figure 1B) indicated that sufficient soil water content was essential for successful discharge of a charged conductor to a grounded conductor through a plant body (data not shown). Based on these results, we used potted plants that had been sufficiently watered for our discharge-exposure assays involving plant shoots.

**Figure 4:**
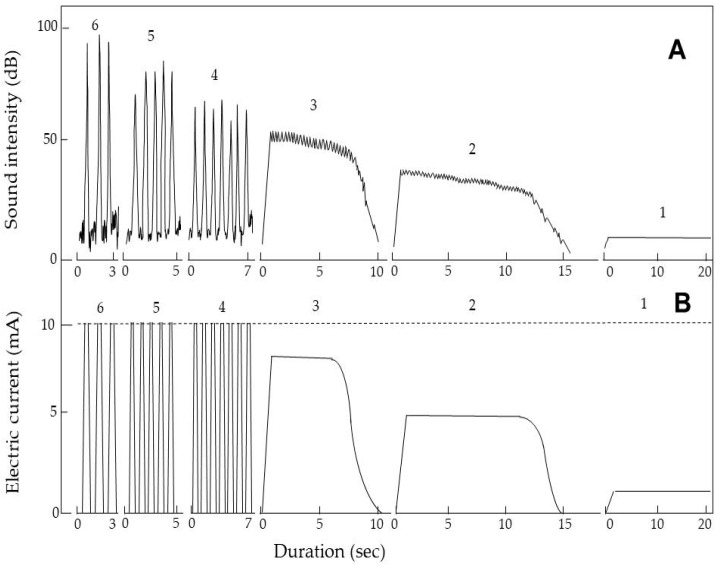
Sound spectrogram (**A**) and electric current profile (**B**) in parallel with electric discharge exposure. Numbers (1–6) in the figure represent the distance (mm) between the negatively charged iron plate and the plant shoot that was subjected to arc-discharge exposure. Intermittent arc discharge occurred at 4–6 mm, continuous arc discharge occurred at 2 and 3 mm, and silent discharge occurred at 1 mm. The magnitude of the electric current resulting from intermittent arc discharge was not measured because the current immediately exceeded the detection limit (10 mA).

Using a discharge-generation method (Figure 1C), we examined the change in the discharge formed in the space between the charged plate and a plant shoot, according to the different distances between these components. Figure 4A,B shows a sound spectrogram and a current profile in parallel with discharge generation, respectively. The arc discharge first occurred when the charged plate was brought within 6 mm of the shoot. At that point, the arc-discharge exposure generated the corresponding intermittent impact sound three times (Video S1 with intermittent arc-discharge sound). As the distance became shorter (5 and 4 mm), the sound intensity decreased. However, the numbers of arc-discharge exposures increased. The electric current of the arc discharges was not estimated because the current immediately exceeded the detection limit. These arc-discharge exposures were sufficiently destructive to propel the apical region away from the shoot stem as a result of the strong discharge impact force (Video S1).

**Figure 5:**
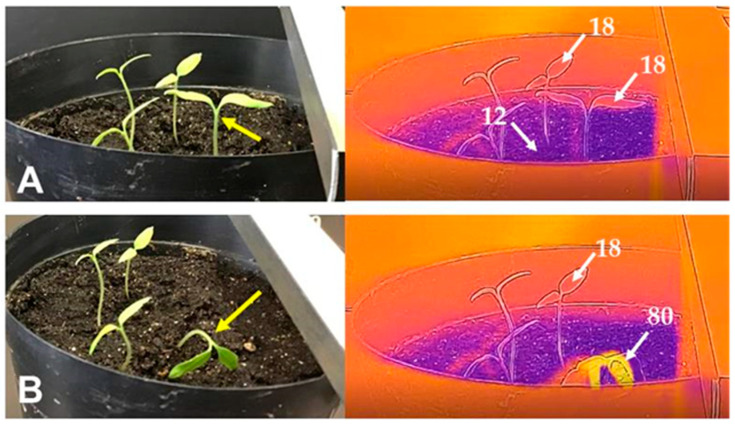
Thermographic detection of arc-discharge-exposed (yellow arrow) and non-exposed shoots of test plants (tomato) sown in pot soil before (**A**) and after arc-discharge exposure at −12 kV charge (**B**). Figures in the thermograms represent temperature (°C) measured with a spot temperature meter of a camera. White arrow represents the site of temperature measurement. Note that the arc-discharge-exposed shoot exhibited the highest temperature color (yellow).

At a distance of 3 mm, the type of discharge changed from intermittent to continuous, as demonstrated by a continuous electric current with a continuous sound (Video S2 with continuous arc-discharge sound). At a distance of 2 mm, both current magnitude and sound intensity decreased, while the duration of discharge exposure increased. At both distances, arc-discharge exposures spontaneously stopped when the apical tip of the shoot had been destroyed. Figure 5 shows thermographic images of the shoots, which reveal differences between the arc-discharge-exposed and non-exposed shoots. The apical area of the arc-discharge-exposed shoot showed the highest temperature zone, indicating that this zone was heated through arc-discharge exposure. These results imply that the heating evaporated water from the shoot, thereby reducing or abolishing the conductivity of the destroyed region of the shoot. At a distance of 1 mm, a soundless electric current of much lower magnitude (i.e., silent discharge) continued until the cessation of voltage application. The silent discharge exposure was harmless and the shoots exhibited continuous elongation. These results strongly imply that arc-discharge exposure destroyed the apical region of the plant shoot as a result of the strong force of impact and/or current-flow-mediated heating, depending on the distance between the shoot and the charged conductor.

Table 1 shows the percentages of test plant shoots with or without elongation after they had been subjected to arc-discharge exposure. The shoots of dicotyledonous (tomato, soybean, watermelon, and sunflower) and monocotyledonous (barley and oat) plants showed distinct features after their tip regions had been destroyed by the arc-discharge exposure treatment. The dicotyledonous plants showed no subsequent shoot elongation (NE-shoots in Table 1), due to disintegration of the growing point and cotyledonal tissues involved in the discharge-exposed apical region. In contrast, the monocotyledonous plants first developed coleoptile tissue, which was destroyed by the exposure treatment. The growing point at the lower position of the shoot remained alive, such that these shoots (RE-shoots) exhibited continuous elongation, despite damage to their apical regions. However, if RE-shoots were kept in the same electric field, subsequent discharge exposures destroyed the newly developed region (below the destroyed tip region) of the shoot. Eventually, repeated damage to the newly developed area caused the death of the shoot. Based on these results, we selected the combination of 6-mm distance and −12-kV charge for the EWE configuration. We presumed that the charged plate could cause arc-discharge exposure to all shoots that emerged inside the area 6 mm from the plate, and that the EWE could cover all plant shoots that emerged between plates with an interval of 12 mm. In practice, the EWE responded to plant shoots that emerged in the central 8 mm area between the plates, because each plate had a mortised insulator rod on its bottom side (Figure 1E).

### 3.2. Flies Can Be Controlled by an Identical Electric-Field-Based Technique

A potential mode of *E. coli* O157 dissemination in the environment is by means of houseflies associated with animal feces and manure. *E. coli* O157 can survive in manure and be transmitted to soil during fertilization with manure [4]. Housefly larvae develop in animal feces and very large populations accumulate, both on cattle farms and in other agricultural facilities [3]. *E. coli* O157 ingested by houseflies remains viable in fly excreta; consequently, houseflies are able to carry and disseminate *E. coli* for several days [3]. Contamination of cultivated and postharvest crops with this pathogen is a serious problem that can endanger the food supply chain [24,25,26]. Accordingly, the present study aimed to develop a method to control houseflies emerging from soil beds before they come into contact with crops in a greenhouse. For this purpose, we developed an additional apparatus that could be operated using the same electrostatic principle, thus enabling integration into the EWE described above. Insects are conductive, so they can receive electric discharges in a dynamic electric field [27,28,29,30]. The focus of the present experiment was the production of a dynamic electric field in which flies were subjected to destructive impact through arc-discharge exposure [21].

In the EWE, all iron plates were negatively charged, such that there was no mechanical discharge between the plates, despite voltage application. Importantly, the target of discharge exposure was grounded plants. The experiment involving houseflies required a ground-to-ground circuit involving a dynamic electric field for arc discharge, because the houseflies were not grounded. This circuit was constructed by pairing two iron plates that were charged and grounded, respectively, as shown in Figure 1G. Importantly, mechanical discharge is expected to occur between the plates, depending on the voltage applied and the distance between the plates [17]. In the present apparatus, the distance was fixed at 12 mm. Our investigation showed that mechanical discharge occurred at −10.1 kV. Therefore, subsequent experiments were conducted using the voltage range (−1 to −10 kV) that caused no mechanical discharge.

These plates were the arc-discharge-generating instrument, but did not necessarily require mechanical arc discharge. When insects entered the electric field between the plates, regardless of location, the insects effectively became intermediate poles. Therefore, the insects were subjected to an arc discharge from the negatively charged plate due to their conductive nature [30]. Accordingly, the electricity was transferred to the insect and then to the grounded plate by means of two-step arc-discharge generation (Figure 1G). This type of discharge has been referred to as insect-mediated arc discharge [21]. The discharge was transient, but sufficiently strong to cause insect death.

Table 2 shows the relationship between the voltage applied and the occurrence of arc-discharge exposure. The insect-mediated arc discharge was first detected at −5 kV. With application of increasing voltage, the percentage of arc-discharge-exposed flies increased; at <−7 kV, all flies were exposed to the arc discharge. Table 2 also shows the lethality rate and intensity of the arc-discharge sound. The increased number of arc-discharge-exposed flies was reflected by the increased percentage of killed flies. Furthermore, larger voltages produced a larger arc-discharge sound. This implies that larger voltages imparted a stronger impact force to the flies. Notably, the percentage of flies killed instantaneously increased as the applied voltage increased (Video S3A with arc-discharge sound). Based on these results, we constructed the EIE with a distance of 12 mm between the plates and a charge of −10 kV to kill adult houseflies emerging from the soil.

### 3.3. Weeds and Houseflies Can Be Simultaneously Controlled Using Electric Discharge Exposure Techniques

An important problem remained unsolved that would affect practical use of the EIE/EWE. Namely, the discharge-exposed targets seemed to revive from apparent death. In the case of weeds, their locations did not change, so they received continuous discharge until death occurred. However, the houseflies were sometimes expelled from the apparatus by the strong impact of the arc discharge. The revival of these flies poses a potential risk for dissemination of *E. coli* O157, if the revived flies are contaminated with this pathogenic bacterium. Notably, approximately 30% of the arc-discharge-exposed houseflies survived for a considerable time after exposure (Table 2) (Video S3B). Accordingly, we aimed to kill all flies instantaneously. Therefore, a new approach was needed, because the pole distance between the plates was fixed, and the highest voltage available had been used.

In the original electrostatic configuration, the negative charge supplied by the voltage generator accumulated evenly on the surface of all of the charged iron plates [31] and was then released to the fly in a single discharge. We hypothesized that an enlarged plate area would accumulate larger amounts of electricity that would be released with greater impact. This greater discharge impact could be confirmed by a stronger arc-discharge sound intensity. To test this hypothesis, we linked different numbers of EIE/EWEs, as shown in Figure 2B, and propelled the flies towards the linked apparatuses.

**Figure 6:**
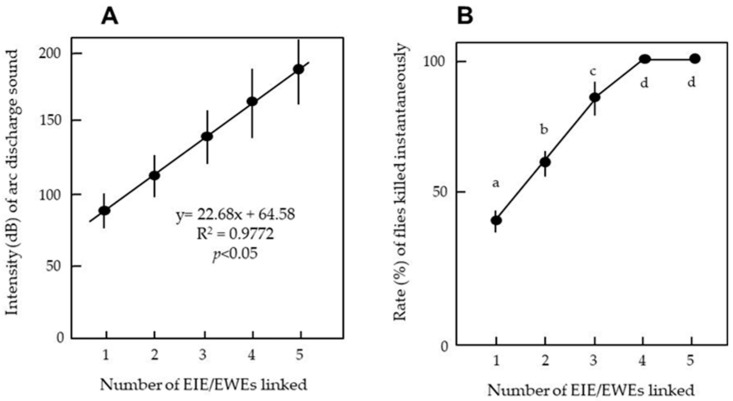
Relationships between the numbers of linked EIE/EWEs and the intensity of the arc-discharge sound (**A**) and the percentage of flies killed instantaneously (**B**). Twenty adult houseflies were used for each apparatus, and the means and standard deviations were calculated from five replicates of the experiments. The letters (^a–d^) indicate significant differences (*p* < 0.05) determined by Tukey’s test.

Figure 6A shows the relationship between the numbers of linked EIE/EWEs and the intensity of the arc-discharge sound generated when a fly was propelled into the electric field of the EIE. Linear regression indicated that the force generated by the arc discharge became stronger in direct proportion to the number of linked apparatuses. Furthermore, the number of flies killed immediately increased during this experiment, reaching a peak (100%) when three EIE/EWEs were linked (Figure 6B). These results clearly support our hypothesis.

The practicality of the EIE/EWE was surveyed during continuous operation under greenhouse conditions. All linked apparatuses responded equivalently to plant shoots irregularly emerging from soil or to adult houseflies irregularly emerging from pupae; eventually, all plant shoots and houseflies were eradicated by the system (Table 3).

In our work to support small farmers in the suburban area of a large city, technical guidance is a primary activity. Notably, the present study was planned in response to the needs of these farmers. Our fundamental aim is to introduce new technical methods that these farmers can implement. The framework of the apparatus is simple and easy to fabricate with ubiquitous metal materials. However, the voltage generator must be purchased. The voltage generator used can be operated by a 12-volt storage battery. It can boost the initial voltage (12 V) to the desired voltage (1–30 kV) using a transformer (coil) and a Cockcroft circuit integrated into an electric circuit in the voltage generator [32]. Two configurations of the voltage generator are commercially available: voltage-fixed and voltage-adjustable. During our investigations, the voltage-adjustable model was used to examine the relationship between the applied voltage and the occurrence of electrostatic phenomena [33]. The voltage-fixed model was used in later experiments, due to its lower cost.

## 4. Conclusions

Electrostatic engineering provides an opportunity for the application of electric-field-based phenomena to the unique context of pest control. In this study, agricultural nuisances emerging from ground soil were targeted for eradication by means of a non-agrochemical method during greenhouse organic farming. The electrostatic phenomenon used for pest control was an electric discharge in a dynamic electric field. The technical viewpoint of the work was the design of a practical apparatus with a single configuration that could be applied to multiple targets. The electric field was created by two parallel metal iron plates: one was linked to a direct current voltage generator to supply a negative charge to the plate, while the other was linked to a grounded line to achieve positive polarization via electrostatic induction. An electric field formed between the opposite charges of the plates. Non-insulated plates produce a dynamic electric field whereby electricity moves through arc discharge. Based on this electrostatic principle, two types of electrostatic apparatuses (EWE and EIE) were fabricated to expose weeds and flies to arc discharge upon entry to the dynamic electric field. The discharge exposure was sufficiently effective that the discharge-imposed targets were killed immediately or after repeated exposure treatment. By unifying these apparatuses, a practical system (EIE/EWE) was constructed for simultaneous control of weeds and flies emerging from ground soil. This apparatus remained functional during long-term operation under greenhouse conditions. The structure of the apparatus was simple and it was constructed easily. Thus, this study provides a practical physical method to control weeds and flies emerging from ground soil, which could be useful for organic farmers.

## Figures and Tables

**Table 1:** Subsequent elongation of plant shoots exposed to arc discharge from iron plates with a −12 kV charge.

Plants Used	Kind of Discharge Used for Exposure	Percentage of		
		Injured Shoots ^III^	NE-Shoots ^IV^	RE-shoots ^V^
tomato	Brush-like corona discharge ^I^	100	96.0 ± 2.2 ^a^	4.0 ± 2.2 ^a^
soybean		100	95.0 ± 3.5 ^a^	5.0 ± 3.5 ^a^
watermelon		100	94.0 ± 4.2 ^a^	6.0 ± 4.2 ^a^
sunflower		100	93.0 ± 2.7 ^a^	7.0 ± 2.7 ^a^
oat		100	3.0 ± 2.7 ^b^	97.0 ± 2.7 ^b^
barley		100	4.0 ± 2.2 ^b^	96.0 ± 2.2 ^b^
tomato	Arc discharge ^II^	100	97.0 ± 2.7 ^a^	3.0 ± 2.7 ^a^
soybean		100	96.0 ± 2.2 ^a^	4.0 ± 2.2 ^a^
watermelon		100	94.0 ± 2.2 ^a^	6.0 ± 2.2 ^a^
sunflower		100	95.0 ± 3.5 ^a^	5.0 ± 3.5 ^a^
oat		100	5.0 ± 3.5 ^b^	95.0 ± 3.5 ^b^
barley		100	4.0 ± 2.2 ^b^	96.0 ± 2.2 ^b^

^I^ Discharge generated at positions 2 and 3 mm from the charged plate. ^II^ Discharge generated at positions 4–6 mm from the charged plate. ^III^ Detected immediately after discharge exposure. ^IV^ Shoots without elongation (at 1 week after discharge exposure). ^V^ Shoots that showed elongation (at 1 week after discharge exposure). Plant shoots were grown in a pot of soil, the bottom of which was covered by a grounded metal net. They were subjected to discharge exposure from the charged iron plate at different distances between the plate and the shoot. Twenty shoots were used for each species and each distance, and the means and standard deviations were calculated from five replicates of the experiments. The letters (^a, b^) in each vertical column indicate significant differences (*p* < 0.05) determined by the Tukey’s test.

**Table 2:** Eradication of houseflies by exposure to arc discharge from iron plates negatively charged with different voltages ^I^.

Voltage (kV) Applied	Percentage of Flies Subjected to Arc Discharge	Percentage of Arc Discharge-Exposed Flies			Lethal Rate (%)	Intensity (dB) of Arc Discharge Sound
		Dead Instantly ^II^	Alive and then Dead ^III^	Alive ^III^		
1	0	n.d.	n.d.	n.d.	0	n.d. ^IV^
2	0	n.d.	n.d.	n.d.	0	n.d.
3	0	n.d.	n.d.	n.d.	0	n.d.
4	0	n.d.	n.d.	n.d.	0	n.d.
5	24.0 ± 9.6 ^a^	5.0 ± 3.5 ^a^	0	95.0 ± 3.5 ^a^	5.0 ± 3.5 ^a^	59.8 ± 5.6 ^a^
6	58.0 ± 9.1 ^b^	21.0 ± 8.9 ^b^	28.0 ± 8.4 ^b^	51.0 ± 2.2 ^b^	49.0 ± 2.2 ^b^	73.6 ± 7.9 ^b^
7	94.0 ± 6.5 ^c^	26.0 ± 2.2 ^b^	62.0 ± 5.7 ^c^	12.0 ± 5.7 ^c^	88.0 ± 5.7 ^c^	77.1 ± 7.3 ^b^
8	100 ^d^	39.0 ± 5.5 ^c^	61.0 ± 5.5 ^c^	0	100 ^d^	94.5 ± 6.6 ^c^
9	100 ^d^	41.0 ± 7.4 ^c^	59.0 ± 7.4 ^c^	0	100 ^d^	96.5 ± 7.5 ^c^
10	100 ^d^	43.0 ± 5.7 ^c^	57.0 ± 5.7 ^c^	0	100 ^d^	99.9 ± 7.9 ^c^

^I^ A pair of iron plates formed an electric field by linking one plate to a voltage generator to supply a negative charge, while the other was linked to a ground line. Adult flies were propelled into the electric field between the plates. ^II,III^ Assessed immediately and at 6 h after discharge-exposure treatment, respectively. ^IV^ Not detected. Twenty shoots were used for each voltage, and the means and standard deviations were calculated from five replicates of the experiments. The letters (^a–d^) indicate significant differences (*p* < 0.05) determined by Tukey’s test.

**Table 3:** Survey of triplicate EIE/EWEs for their ability to simultaneously control plant shoots and adult houseflies emerging from soil under greenhouse conditions.

Target of Discharge Exposure	Site	Days		Target of Discharge Exposure	Site	Days	
		I ^w^	II ^x^			III ^y^	IV ^z^
Dicots	A	5.1 ± 0.7 ^a^	1.1 ± 0.4 ^a^	Houseflies	A	5.6 ± 0.5 ^a^	0.7 ± 0.2 ^a^
	B	4.8 ± 0.8 ^a^	1.4 ± 0.5 ^a^		B	5.4 ± 0.8 ^a^	0.5 ± 0.3 ^a^
	C	5.2 ± 0.6 ^a^	1.3 ± 0.5 ^a^		C	5.9 ± 0.7 ^a^	0.5 ± 0.2 ^a^
Monocots	A	4.2 ± 0.5 ^a^	5.1 ± 0.9 ^b^				
	B	4.5 ± 0.6 ^a^	6.8 ± 0.8 ^b^				
	C	5.1 ± 0.8 ^a^	6.3 ± 0.6 ^b^				

^w^ Days required for shoots to reach the electric field. ^x^ Days required to kill all shoots after they had reached the electric field. ^y^ Days required for pupae to generate adult flies. ^z^ Days required to kill adult flies after emergence. The means and standard deviations were calculated from three replicates of the experiments. The letters (^a, b^) in each vertical column indicate significant differences (*p* < 0.05) determined by Tukey’s test.

## Supplementary Materials

The following are available online at https://www.mdpi.com/2075-4450/11/12/861/s1, Video S1: Exposure of plant shoots (tomato) to intermittent arc discharge, accompanied by 2–6 intermittent sounds (charge, −12 kV; distance, 4–6 mm), Video S2: Exposure of plant shoots (tomato) to continuous arc discharge, accompanied by continuous sound (charge, −12 kV; distance, 3 mm), Video S3: Exposure of adult houseflies to arc discharge, accompanied by 1–2 sounds (charge, −10 kV). Flies died immediately (A) or were alive and then dead (B) after the arc discharge exposure treatment.

## References

[1] Russell J.B., Jarvis G.N. (2001). Practical mechanisms for interrupting the oral-fecal lifecycle of *Escherichia coli*. Mol. Microbiol. Biotechnol..

[2] Ahmad A., Nagaraja T.G., Zurek L. (2007). Transmission of *Escherichia coli* O157:H7 to cattle by house flies. Prev. Vet. Med..

[3] Alam M.J., Zurek L. (2004). Association of *Escherichia coli* O157:H7 with houseflies on a cattle farm. Appl. Environ. Microbiol..

[4] Mukherjee A., Cho S., Scheftel J., Jawahir S., Smith K., Diez-Gonzalez F. (2006). Soil survival of *Escherichia coli* O157:H7 acquired by a child from garden soil recently fertilized with cattle manure. J. Appl. Microbiol..

[5] Matsuda Y., Ikeda H., Moriura N., Tanaka N., Shimizu K., Oichi W., Nonomura T., Kakutani K., Kusakari S., Higashi K. (2006). A new spore precipitator with polarized dielectric insulators for physical control of tomato powdery mildew. Phytopathology.

[6] Tanaka N., Matsuda Y., Kato E., Kokabe K., Furukawa T., Nonomura T., Honda K., Kusakari S., Imura T., Kimbara J. (2008). An electric dipolar screen with oppositely polarized insulators for excluding whiteflies from greenhouses. Crop Prot..

[7] Matsuda Y., Nonomura T., Kakutani K., Takikawa Y., Kimbara J., Kasaishi Y., Kusakari S., Toyoda H. (2011). A newly devised electric field screen for avoidance and capture of cigarette beetles and vinegar flies. Crop Prot..

[8] Nonomura T., Matsuda Y., Kakutani K., Kimbara J., Osamura K., Kusakari S., Toyoda H. (2012). An electric field strongly deters whiteflies from entering window-open greenhouses in an electrostatic insect exclusion strategy. Eur. J. Plant Pathol..

[9] Kakutani K., Matsuda Y., Nonomura T., Kimbara J., Kusakari S., Toyoda H. (2012). Practical application of an electric field screen to an exclusion of flying insect pests and airborne conidia from greenhouses with a good air penetration. J. Agric. Sci..

[10] Takikawa Y., Matsuda Y., Nonomura T., Kakutani K., Okada K., Shibao M., Kusakari S., Miyama K., Toyoda H. (2020). Exclusion of whiteflies from a plastic hoop greenhouse by a bamboo blind-type electric field screen. J. Agric. Sci..

[11] Takikawa Y., Matsuda Y., Kakutani K., Nonomura T., Kusakari S., Okada K., Kimbara J., Osamura K., Toyoda H. (2015). Electrostatic insect sweeper for eliminating whiteflies colonizing host plants; a complementary pest control device in an electric field screen-guarded greenhouse. Insects.

[12] Toyoda H., Kusakari S., Matsuda Y., Kakutani K., Xu L., Nonomura T., Takikawa Y. (2019). Practical applications of earth net-free electric field screen. An Illustrated Manual of Electric Field Screens: Their Structures and Functions.

[13] Otsu Y., Matsuda Y., Shimizu H., Ueki H., Mori H., Fujiwara K., Nakajima T., Miwa A., Nonomura T., Sakuratani Y. (2003). Biological control of phytophagous ladybird beetles *Epilachna vigintioctopunctata* (Col., Coccinellidae) by chitinolytic phylloplane bacteria *Alcaligenes paradoxus* entrapped in alginate beads. J. Appl. Ent..

[14] Otsu Y., Mori H., Komuta K., Shimizu H., Nogawa S., Matsuda Y., Nonomura T., Sakuratani Y., Tosa Y., Mayama S. (2003). Suppression of leaf feeding and oviposition of phytophagous ladybird beetles *Epilachna vigintioctopunctata* (Coleoptera: Coccinellidae) by chitinase gene-transformed phylloplane bacteria and their specific bacteriophages entrapped in alginate gel beads. J. Econ. Entomol..

[15] Otsu Y., Matsuda Y., Mori H., Ueki H., Nakajima T., Fujiwara K., Matsumoto M., Azuma N., Kakutani K., Nonomura T. (2004). Stable Phylloplane Colonization by entomopathogenic bacterium *Pseudomonas fluorescens* KPM-018P and biological control of phytophagous ladybird beetles *Epilachna vigintioctopunctata* (Coleoptera: Coccinellidae). Biocontrol Sci. Technol..

[16] Bizzi C.A., Barin J.S., Hermes A.L., Mortari S.R., Flores É.M.M. (2011). A fast microwave-assisted procedure for loss on drying determination in saccharides. J. Braz. Chem. Soc..

[17] Halliday D., Resnick R., Walker J., Johnson S., Ford E. (2005). Electric discharge and electric fields. Fundamentals of Physics.

[18] Kaiser K.L. (2006). Air breakdown. Electrostatic Discharge.

[19] Nonomura T., Matsuda Y., Kakutani K., Takikawa Y., Toyoda H. (2008). Physical control of powdery mildew (*Oidium neolycopersici*) on tomato leaves by exposure to corona discharge. Can. J. Plant Pathol..

[20] Jones E., Childers R. (2002). Electric current and resistance. Physics.

[21] Matsuda Y., Takikawa Y., Nonomura T., Kakutani K., Okada K., Shibao M., Kusakari S., Miyama K., Toyoda H. (2018). Selective electrostatic eradication of *Sitopholus oryzae* nesting in stored rice. J. Food Technol. Pres..

[22] Kakutani K., Matsuda Y., Takikawa Y., Nonomura T., Okada K., Shibao M., Kusakari S., Miyama K., Toyoda H. (2018). Electrocution of mosquitoes by a novel electrostatic window screen to minimize mosquito transmission of Japanese encephalitis viruses. Int. J. Sci. Res..

[23] Fessas D., Schiraldi A. (2001). Water properties in wheat flour dough I: Classical thermogravimetry approach. Food Chem..

[24] Ibekwe A.M., Grieve C.M., Papiernik S.K., Yang C.-H. (2009). Persistence of *Escherichia coli* O157:H7 on the rhizosphere and phyllosphere of lettuce. Lett. Appl. Microbiol..

[25] Brandl M.T. (2008). Plant lesions promote the rapid multiplication of *Escherichia coli* O157:H7 on postharvest lettuce. Appl. Environ. Microbiol..

[26] Luo Y., He Q., McEvoy J.L. (2010). Effect of storage temperature and duration on the behavior of *Escherichia coli* O157:H7 on packaged fresh-cut salad containing romaine and Iceberg lettuce. J. Food Sci..

[27] Kakutani K., Matsuda Y., Haneda K., Nonomura T., Kimbara J., Kusakari S., Osamura K., Toyoda H. (2012). Insects are electrified in an electric field by deprivation of their negative charge. Ann. Appl. Biol..

[28] Kakutani K., Matsuda Y., Haneda K., Sekoguchi D., Nonomura T., Kimbara J., Osamura K., Kusakari S., Toyoda H. (2012). An electric field screen prevents captured insects from escaping by depriving bioelectricity generated through insect movements. J. Electrostat..

[29] Nonomura T., Matsuda Y., Kakutani K., Kimbara J., Osamura K., Kusakari S., Toyoda H. (2014). Electrostatic measurement of dischargeable electricity and bioelectric potentials produced by muscular movements in flies. J. Electrost..

[30] Takikawa Y., Takami T., Kakutani K. (2020). Body water-mediated conductivity actualizes the insect-control functions of electric fields in houseflies. Insects.

[31] Giancoli D.C., Challice J. (2005). Electric charge and electric field. Physics, Principles with Applications.

[32] Wegner H.E., Geller E., Moore K., Weil J. (2002). Electrical charging generators. McGraw-Hill Encyclopedia of Science and Technology.

[33] Kusakari S., Okada K., Shibao M., Toyoda H. (2020). High voltage electric fields have potential to create new physical pest control systems. Insects.

